# Measurement of catestatin and vasostatin in wild boar *Sus scrofa* captured in a corral trap

**DOI:** 10.1186/s13104-021-05742-1

**Published:** 2021-08-30

**Authors:** Åsa Fahlman, Johan Lindsjö, Ulrika A. Bergvall, Erik O. Ågren, Therese Arvén Norling, Mats Stridsberg, Petter Kjellander, Odd Höglund

**Affiliations:** 1 SLU Swedish Biodiversity Centre, Department of Urban and Rural Development, Swedish University of Agricultural Sciences (SLU), 750 07 Uppsala, Sweden; 2 Department of Animal Environment and Health, SLU, 750 07 Uppsala, Sweden; 3 Grimsö Wildlife Research Station, Department of Ecology, SLU, 739 93 Riddarhyttan, Sweden; 4 Department of Pathology and Wildlife Diseases, National Veterinary Institute, 751 89 Uppsala, Sweden; 5 Department of Organismal Biology, Genome Engineering Zebrafish, SciLifeLab, Uppsala University, 752 36 Uppsala, Sweden; 6 Department of Medical Sciences, Uppsala University, 751 85 Uppsala, Sweden; 7 Department of Clinical Sciences, SLU, 750 07 Uppsala, Sweden

**Keywords:** Animal welfare, Catestatin, CgA, Live-trap capture, Stress, Trapping, Vasostatin, 3Rs

## Abstract

**Objective:**

Our aim was to analyse the chromogranin A-derived peptides vasostatin and catestatin in serum from wild boar (*Sus scrofa*) captured in a corral trap. Acute capture-related stress quickly leads to a release of adrenalin and noradrenalin, but these hormones have a short half-life in blood and are difficult to measure. Chromogranin A (CgA), a glycoprotein which is co-released with noradrenalin and adrenalin, is relatively stable in circulation and the CgA-derived peptides catestatin and vasostatin have been measured in domestic species, but not yet in wildlife.

**Results:**

Vasostatin and catestatin could be measured and the median (range) serum concentrations were 0.91 (0.54–2.86) and 0.65 (0.35–2.62) nmol/L, respectively. We conclude that the CgA-derived peptides vasostatin and catestatin can be measured in wild boar serum and may thus be useful as biomarkers of psychophysical stress.

## Introduction

Physiological alterations can be strong indicators of capture-related stress in wild animals [1–4]. Stress during live-trap capture of wild animals may alter several physiological blood variables [5, 6] and various trap methods can affect physiological variables differently [6–9]. In a study that assessed multiple haematological and biochemical values in immobilised wild boar, the results indicated that capture in drop nets, corral and cage traps were more stressful than darting with blow pipe without previous physical capture [9]. In another study, lactate and glucose were higher in wild boar captured and immobilised in corral traps than in cage traps [10]. Acute stress quickly leads to a release of adrenalin and noradrenalin, but these hormones have a very short half-life in blood and are difficult to measure in situ [11]. In contrast, chromogranin A (CgA), a glycoprotein which is co-released with noradrenalin and adrenalin at a stressful event, is relatively stable in circulation. The CgA-derived peptides catestatin and vasostatin can be measured in serum, plasma, or saliva [12, 13]. Chromogranin A has been used for evaluation of stress response in several domestic species [14–21]*.* In domestic pigs, salivary CgA has been used as a biomarker of stress in different situations, such as immobilization with a nasal snare [15], after refeeding following a period of food deprivation [22] and after isolation or regrouping [23].

Analysis of the CgA-derived peptides catestatin and vasostatin has not previously been reported in a wildlife species. Potentially, CgA can be used for evaluation of stress related to various capture methods. The aim of this study was to analyse concentrations of catestatin and vasostatin in serum samples from wild boar that were euthanized after live trapping.

## Main text

### Materials and methods

Live-trap capture of free-ranging wild boar in a corral-style trap (JP BUR, Oskarström, Sweden) was conducted from 11 March to 21 April, 2015, at Wij Säteri, Bålsta, Sweden (Lat: 59.59, Long: 17.43). The time from when the trap was set until one or several wild boar were captured ranged from 62 to 206 min and the total time wild boar spent in the trap ranged from ~ 2.5–12.4 h. Further details of the capture methodology have been described by Fahlman et al. [24]. The captures were conducted as part of an assignment from the Swedish Environmental Protection Agency (SEPA) to the Swedish University of Agricultural Sciences (SLU), Department of Ecology at Grimsö Wildlife Research Station, to evaluate new live-traps for wildlife capture before approval as new hunting methods in Sweden. Approval of a new trap construction is based on gross necropsy findings of 20 trapped and euthanised animals. Live-trap capture of wild boar followed by killing inside the trap by gunshot is a recently introduced hunting method in Sweden, and these captures were conducted during the evaluation period, before approval of this trap for hunting. Ethical approval to test the corral-style trap by capture of free-ranging wild boar and subsequent euthanasia of up to 20 subadults was given by the Ethical Committee on Animal Research, Uppsala, Sweden (C122/13). The wild boar were euthanized in the corral-style trap by gunshot to the brain (0.22 LR cartridge used in a revolver or a rifle) by the wildlife manager that conducted all captures. Euthanasia was conducted as soon as practically possible upon arrival at the trap, to minimize the time the wild boar were exposed to human presence. The wildlife manager was standing right outside the trap when firing the shots at a maximum shot distance of 4 m from the wild boar. This is the method for killing wild boar captured in this trap when it is used for hunting. The time from arrival of the wildlife manager until all animals were euthanized ranged from 1.6 to 11.1 min for group captures and 0.7–1.6 min for single captures. The evaluation of animal welfare during live-trap capture was based mainly on pathological examinations, as specified by SEPA [25], which required euthanasia. For improved animal welfare evaluation, we studied wild boar behaviour [24] in conjunction with the captures conducted within the SEPA assignment. In addition, we also collected blood samples for this study. Thus, no wild boar was captured solely for the purpose of blood sampling or behavioural assessment. This contributes to *the principle of the 3Rs* through *reduction* and promotes future *refinement* since evidence-based knowledge on physiological and behavioural alterations during the capture process can lead to improved trapping methods.

#### Blood sample collection and analysis

For analysis of the CgA-derived peptides catestatin and vasostatin, blood samples were collected post-mortem from a cut in the jugular vein of 16 subadult wild boar immediately after euthanasia. The blood samples were centrifuged within 24 h, and serum was separated and stored in cryovials in − 20 °C at the National Veterinary Institute (SVA), Uppsala, Sweden. We stored the serum samples for 3–9 weeks until analysis on 15 May 2015 at the Clinical Chemistry Laboratory, Uppsala University Hospital, Uppsala, Sweden. Competitive radioimmunoassays (RIA) were used (vasostatin, CGA 17–38, and catestatin, CGA 361–372), as described by Stridsberg et al. [18]. All samples were analysed in duplicates.

#### Statistical analysis

Data was log-transformed (ln) to conform more closely to the normal distribution. Analysis for Pearson’s correlation coefficient was performed in JASP Team (Version 0.14.1, Computer software, 2020) to determine if there was a correlation between the catestatin and vasostatin values. The significance level was set to 0.05.

### Results

**Fig. 1:**
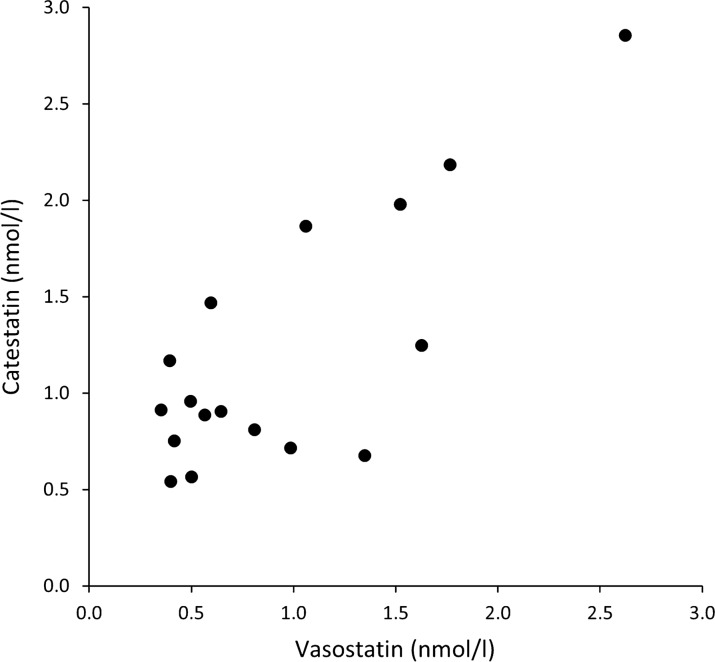
Serum concentrations of catestatin and vasostatin in 16 wild boar captured in a corral trap

The CgA-derived peptides catestatin and vasostatin were measurable in serum samples from wild boar. The median (range) for catestatin and vasostatin levels were 0.91 (0.54–2.86) and 0.65 (0.35–2.62) nmol/L, respectively (Fig. 1). There was a significant correlation between catestatin and vasostatin values (log-transformed) (Pearson’s r = 0.669, n = 16, P = 0.005).

### Discussion

We report measurements of the CgA-derived peptides catestatin and vasostatin in wild boar, of potential use as biomarkers of psychophysical stress in wild animals. Chromogranin A and its derivatives are used as diagnostic and prognostic markers for various diseases and as a biomarker of psychophysical stress in humans and domestic species, such as pigs, horses, donkeys and dogs [12, 13, 17, 19, 20, 26–28]. To the best of our knowledge, this is the first report on measurements of catestatin and vasostatin in a wild mammalian species. The upper ranges of the catestatin and vasostatin concentrations in the wild boar serum were 2.86 nmol/l and 2.62 nmol/l, respectively. In domestic pig serum, similar vasostatin concentrations have been measured (mean 2.3 ± SE 0.3 nmol/l, n = 5) [14], whereas catestatin has not been reported. Interestingly, in comparison to values measured in dogs with minimal stress behaviour during blood sampling (catestatin range 0.53–0.98 nmol/l, vasostatin range 0.11–1.30 nmol/l) [29], our highest wild boar values were more than twice as high which may reflect capture stress. Behavioural alterations indicative of capture-induced stress were documented through filming of the study animals, which has been published elsewhere [24]. To further increase our understanding of CgA as a biomarker of stress in wild boar, samples also need to be collected from animals subjected to different levels of stress and from animals that are not stressed.

The catestatin and vasostatin concentrations in the wild boar serum correlated, which contrasts with a previous study involving healthy dogs [29]. However, comparisons should be done cautiously as the dog study included analysis of saliva and different statistical methods were used. Furthermore, there may be differences between species. The plasma concentration of catestatin and vasostatin reflect both the intact CgA molecule and the two degradation derived peptides, which may have different clearance rates [30].

Physiological alterations may result in adverse effects on an animal’s short- and long-term welfare and survival [8, 31]. Monitoring stress using physiological indicators allows the comparison and evaluation of different capture techniques [9, 32]. Cortisol concentrations, haematological and biochemical variables have been measured for assessment of stress and animal welfare for wild boar captured in cage traps [5, 9], corral traps, drop nets and by darting [9]. Further, the cortisol response in wild boar and four other ungulate species (moose, red deer, fallow deer, roe deer) has been reported in relation to various traumatic situations and hunting methods. Interestingly, cortisol levels were 5–10 times higher in wild boar than in the other ungulate species [33], indicating physiological differences between species. Ideally, a panel of various biomarkers and multiple haematological and chemistry variables should be used to evaluate the stress response [5, 6, 10, 26], which unfortunately was not possible in the present study due to limited funding. Biomarkers that can be used for evaluation of stress in domestic pigs, such as cortisol, CgA, and immunoglobulin A (IgA), have been reviewed by Martínez-Miró et al. [26]. In domestic pigs, salivary CgA and IgA appeared to be more sensitive stress markers than cortisol and testosterone during isolation from other pigs, which caused a significant increase in exploratory behaviour (sniffing, touching and walking through the pen) and vocalization [23]. Further studies are needed to investigate and potentially validate catestatin and vasostatin as biomarkers of stress in wild boar, through concurrent analysis of multiple physiological blood variables and in comparison to individual behaviour and pathology.

### Conclusion

The CgA-derived peptides vasostatin and catestatin can be measured in wild boar serum and may be useful as biomarkers of psychophysical stress.

### Limitations

The present study included a small sample size. Reference values for vasostatin and catestatin in wild boar serum remain to be determined, which requires a larger sample size.

## Data Availability

The datasets analysed during the current study are available from the corresponding author on reasonable request.

## Abbreviations

CgA: Chromogranin A
SEPA: Swedish Environmental Protection Agency
SLU: Swedish University of Agricultural Sciences
SVA: National Veterinary Institute
IgA: Immunoglobulin A
